# Metformin Use and Clinical Outcomes in Autosomal Dominant Polycystic Kidney Disease: A Nationwide Cohort Study

**DOI:** 10.3390/biomedicines13030635

**Published:** 2025-03-05

**Authors:** I-Ching Kuo, Ming-Yen Lin, Yu-Hsiang Tsao, Yi-Wen Chiu, Jia-Jung Lee

**Affiliations:** 1 Graduate Institute of Clinical Medicine, College of Medicine, Kaohsiung Medical University, Kaohsiung 807017, Taiwan; 980135kmuh@gmail.com; 2 Division of Nephrology, Department of Internal Medicine, Kaohsiung Medical University Hospital, Kaohsiung Medical University, Kaohsiung 807017, Taiwan; mingyenlin3@gmail.com (M.-Y.L.); chiuyiwen@gmail.com (Y.-W.C.); 3 Department of Public Health, College of Health Sciences, Kaohsiung Medical University, Kaohsiung 807017, Taiwan; tsaokmu@gmail.com; 4 Division of Medical Statistics and Bioinformatics, Department of Medical Research, Kaohsiung Medical University Hospital, Kaohsiung 807017, Taiwan; 5 Faculty of Medicine, College of Medicine, Kaohsiung Medical University, Kaohsiung 807017, Taiwan

**Keywords:** metformin, CKD, ESKD

## Abstract

**Background/Objectives**: Autosomal dominant polycystic kidney disease (ADPKD) is a progressive genetic disorder marked by bilateral renal cysts and extrarenal manifestations, ultimately resulting in renal failure. Emerging research indicates that metformin might influence the intracellular mechanisms of ADPKD, though its clinical significance remains uncertain. **Methods**: We applied the Taiwan National Health Insurance Database (NHIRD) to investigate the clinical impact of metformin utilization in ADPKD patients in real-world practice. The metformin user group was defined by more than 90 days of usage. To mitigate selection bias, we established a non-user group with a 1:2 ratio, matching for age, sex, and comorbidities by a propensity score matching method. **Results**: A total of 10,222 ADPKD cases were identified in the NHIRD between 2009 and 2018. After matching, the metformin user group was composed of 778 cases with a mean age of 59.5 ± 13.9 years, and the non-user group of 1546 cases with a mean age of 59.3 ± 14.4 years. The time from the index date to the outcome of ESKD in ADPKD was 5.3 ± 2.2 years in the metformin user group and 5.3 ± 2.3 years in the metformin non-user group, respectively. The metformin user group exhibited a significant reduction in the risk of end-stage kidney disease (ESKD), as indicated in the fully adjusted model (0.75, 95% CI 0.58–0.97, *p* = 0.03). A decreased risk of major adverse cardiovascular events (MACEs) was noted in metformin users, with an adjusted hazard ratio (HR) of 0.78 (95% CI 0.65–0.95, *p* = 0.01). Sensitivity analysis showed similar results by excluding late-stage CKD (CKD stage 5 or erythropoietin-stimulating agents use). **Conclusions**: Metformin usage in real-world practice showed lower hazards of ESKD and MACEs in patients with ADPKD, except for those with advanced CKD.

## 1. Introduction

Autosomal dominant polycystic kidney disease (ADPKD) is a common genetic cause of end-stage kidney disease (ESKD) in adults. Mutations in polycystin 1 (PKD1, 78%), polycystin 2 (PKD2, 15%), and other rarely described genes such as GANAB [1] and DNAJB11 [2] lead to a gradual loss of kidney function. Recent large-population sequencing data indicated a minimal genetic ADPKD prevalence of 9.3 per 10,000 people [3]. However, ADPKD prevalence is much lower in diagnosis-based studies because of the variability of genetic penetrance. For example, a large, racially and ethnically diverse US population had a prevalence of clinically diagnosed ADPKD of 42.6 per 100,000 people [4]. The direct manifestation of ADPKD in the kidney is characterized by multiple cysts throughout the kidney parenchyma. Subsequently, progressive loss of kidney function leads to ESKD over decades. Moreover, the disease is associated with extrarenal manifestation involving hepatic and pancreatic cysts, and cardiovascular abnormalities such as hypertension, cerebral aneurysms, valvular abnormalities, primary cardiomyopathies (dilated or hypertrophic), and arrhythmias [5]. Cardiovascular disease (CVD) is known to be the major cause of death in ADPKD [6].

The pathogenesis of ADPKD is complex and involves genetic defects that promote cyst epithelial cell proliferation, fluid secretion into the cysts, and abnormal cilia function [7]. The PKD gene products, polycystin-1 (PC1) and polycystin-2 (PC2), are integral membrane proteins localized in the primary cilium of renal tubular epithelia and modulate several signaling pathways in cooperation with other proteins. At the molecular level, dysregulated cellular pathways and metabolism, including cyclic adenosine monophosphate (cAMP), mammalian target of rapamycin (mTOR), and Janus kinase/signal transducer and activator of transcription (JAK/STAT) pathways are implicated in PKD [8]. Furthermore, PKD has also been described as a disease of metabolic derangement. There is a shift in energy production to excessive aerobic glycolysis in cyst cells in a murine model of PKD and human kidney cells with a PKD1 mutation [9]. Currently, a vasopressin 2 receptor antagonist (V2RA), Tolvaptan, is the only approved treatment for ADPKD. It slows the rate of increased kidney volume and declining kidney function by suppressing cAMP signaling [10,11,12]. Furthermore, high water intake in patients at risk of rapid progression of ADPKD reduced vasopressin levels and slowed the rate of total kidney volume growth, providing a nonpharmacologic treatment [13].

Metformin, a biguanide derivative, is recommended as the initial treatment for type 2 diabetes mellitus (DM). Metformin has drawn attention as a potential therapy for ADPKD treatment due to its abilities to activate 5′ AMP-activated protein kinase (AMPK) and suppress cystic fibrosis transmembrane conductance regulator (CFTR) and mTOR [14,15,16,17]. Metformin inhibits cystogenesis in mouse and zebrafish models [17,18]. More recently, metformin treatment was found to reduce inflammatory and injury markers, along with cell proliferation markers, in a transgenic ADPKD mouse model [19]. Given the accumulating evidence suggesting metformin’s effects in PKD models, the Trial of Administration of Metformin in PKD (TAME-PKD) was established to test the safety, tolerability, and efficacy of metformin in ADPKD patients with an eGFR of >50 mL/min per 1.73 m^2^. Although the phase 2 trial was not designed to detect efficacy in preventing disease progression, metformin treatment has shown promise in slowing the decline in eGFR over the 2-year study period [20].

Despite promising results from preclinical studies, the clinical efficacy of metformin in ADPKD patients remains unclear. Therefore, this study aims to evaluate the associations of metformin use with clinical outcomes in a national population-based ADPKD cohort.

## 2. Materials and Methods

### 2.1. Data Source

This retrospective study was conducted using data from the National Health Insurance Research Database (NHIRD) from the Health and Welfare Data Science Centre, Ministry of Health and Welfare (No: H109256) in Taiwan. We used the full population dataset of the NHIRD, comprising 99.9% of the population of Taiwan, which contains information about insured patients, including dates of birth, sex, residency area, diagnostic codes, outpatient visits, hospitalizations, procedures, and drug prescriptions. The diagnosis of diseases in the NHIRD was based on the International Classification of Diseases, 9th Revision, Clinical Modification (ICD-9-CM) before 2015 and has been based on the ICD-10-CM since 2016. The NHIRD is mainly used for reimbursement, offering comprehensive data on medication prescriptions and procedure interventions, but it lacks clinical laboratory or examination information. To ensure data safety, the data were anonymized and analyzed only in an arranged isolated office disconnected from the internet. This study was approved by the institutional review board of KMUH, and informed consent was waived (KMUHIRB-E(I)-20190260). All the study procedures were conducted according to the principles of the Declaration of Helsinki.

### 2.2. Study Design and Participants

This study included patients who had a diagnosis of ADPKD identified using a combination of ICD-9-CM (753.12 and 753.13) and ICD-10 codes (Q61.2, Q61.3) in the NHIRD between 1 January 2009 and 1 January 2018. The ADPKD diagnoses were confirmed by at least two records of outpatient visits within one year and/or one diagnosis upon hospitalization during the study period to enhance diagnosis accuracy. Patients who were younger than 20 years old, had incomplete demographic data, had a follow-up duration of less than one year, underwent dialysis, or had a malignancy diagnosis before the index date were excluded. Eligible patients who had taken metformin for more than 90 days after their first prescription were referred to as metformin users, and the remaining participants as non-users. The date of initial diagnosis was defined as the index date. The follow-up period was from the index date to the date of death, the commencement of dialysis, the independent occurrence of any of the study outcomes, or the end date of the study period (31 December 2018), whichever occurred first.

The baseline characteristics were identified before the index date to track any history of comorbidities from the NHIRD. Comorbidities were determined according to ICD-9-CM or ICD-10-CM coding (Supplementary Table S1) as at least one inpatient or two outpatient diagnoses of a given disease within 1 year before the index date. Comorbidity severity was estimated using the Charlson comorbidity index (CCI) [21]. The use of specific medications was identified by inspecting specific prescription drug codes within 90 days before the index date.

### 2.3. Outcomes

The main study outcomes were ESKD, all-cause mortality, and the composite endpoint of major adverse cardiovascular events (MACEs), including acute coronary syndrome, acute ischemic stroke, and acute heart failure (Table S1). ESKD was defined as the initiation of dialysis or renal transplantation. The onset of ESKD patients’ receiving of dialysis was assessed, which was defined as the date of commencing long-term dialysis for at least 90 days with catastrophic illness registration. All-cause mortality was confirmed by the Taiwan Death Registration data. MACEs were identified according to the principal diagnostic codes during hospitalization or emergency department visits, which had been validated previously [22,23]. In the mortality analysis, all patients were followed up until death or 31 December 2018. Due to restrictions on applying for the NHIRD database, the follow-up period of this study was set to 31 December 2018.

### 2.4. Statistical Analysis

The ADPKD patient characteristics of the metformin users and non-users were compared to determine potential confounding factors. Additional analyses in patients who had both DM and ADPKD were conducted to verify the robustness of the associations of metformin therapy on outcomes. That means that we defined the ADPKD + DM group as those patients with confirmed coding of DM who were ADPKD patients to minimize the confounding effects of diabetes on clinical outcome evaluations. Baseline characteristics were compared by the independent *t*-test and χ2 tests to examine the distributions of continuous variables and distributions of categorical variables between the user and the non-user groups, respectively. The propensity score matching method was applied to balance distributions of covariates between metformin users and non-users to optimally reduce confounding and selection bias. The propensity scores were estimated by multiple logistic regression, and matching pairs were constructed using the nearest neighbor algorithm, assuming a 95% to 100% similarity range. Finally, the propensity scores of the metformin user group were matched to the non-user group in 1:1 and 1:2 ratios to obtain the final analysis sample.

The incidence rate was calculated by dividing the total number of study outcomes, including ESKD, MACEs, and all-cause mortality, during the follow-up period by person-years at risk. The cumulative incidence was compared between metformin users and non-users using the Kaplan–Meier estimator with cumulative incidence, and the Log-Rank test was used to examine the differences between the two groups. Cox proportional hazards models were used to compare the outcomes between groups after adjustment for baseline covariates and reported as hazard ratios with 95% confidence intervals (CIs). Age, gender, CCI score, gout, hypertension, index year, dyslipidemia, cardiovascular disease, peripheral artery disease, and cerebrovascular disease were used to adjust for confounders in the multivariable analysis. Since death is the competing risk of ESKD and might affect the hazard estimations, the Cox proportional hazards model was extended to consider death as a competing event. We conducted subgroup analyses to detect modification effects on the main result by stratifying age (<60 years or ≥60 years), sex, CCI, diagnosis of cardiovascular disease, and use of an angiotensin-converting enzyme inhibitor or angiotensin receptor blocker. To assess the robustness of the results, we performed a series of sensitivity analyses. First, we limited the analysis to the study subjects with similar kidney function. Since the baseline eGFR data were not available from the NHIRD, ICD-9 and ICD-10 codes or prescription of an erythropoietin-stimulating agent (ESA) for more than 90 days (based on the NHI regulation of ESA prescription reimbursement: those with eGFR < 15 mL/min/1.73 m^2^ and a hemoglobin (Hb) level < 9 g/dL) were used to identify the most representative patients with stage 5 CKD. Others without a diagnostic code of stage 5 CKD or receiving ESA therapy were considered as other CKD stages, including stages 1 to 4 CKD. Furthermore, we analyzed the dose effect according to the cumulative defined daily dose (DDD) during the 90-day exposure period (≤7 DDD, 7–12 DDD, or >12 DDD), relative to no metformin use. The DDD is defined as the assumed average daily maintenance dose for a drug, which is 2000 mg for metformin according to the World Health Organization. Finally, we also used insulin use as a surrogate for DM severity to assess the influence of the severity of the DM on the main results. A two-tailed *p* value < 0.05 was considered statistically significant, and all statistical analyses were conducted using SAS, version 9.4 (SAS Institute, Cary, NC, USA).

## 3. Results

**Figure 1:**
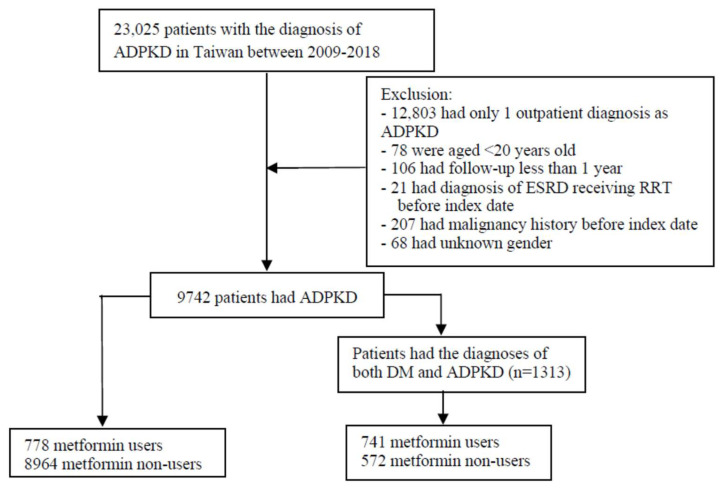
Study design and patient selection flow chart.

A total of 12,803 patients were diagnosed with ADPKD between 1 January 2009 and 1 January 2018 (Figure 1), of which 9742 patients met the inclusion criteria and were enrolled in this study. Among them, 778 patients were metformin users and 8964 were non-users. The baseline characteristics before matching are shown in Table S2. After matching participants in a 1:2 ratio according to propensity score to mitigate selection bias and minimize the confounding effects, the metformin user group consisted of 778 patients and the non-user group consisted of 1546 patients for the outcome analysis. The baseline characteristics of the groups are presented in Table 1. In the matched cohort, all analyzed covariates were balanced between the two groups. The mean ages of the groups were 59.5 ± 13.9 and 59.3 ± 14.4 years, respectively, and both groups had a high prevalence of hypertension and dyslipidemia. Furthermore, 67 (8.6%) metformin users and 260 (16.8%) non-users had defined stage 5 CKD, determined by ICD codes and ESA prescription. The follow-up years were 5.9 ± 3.0 in metformin users and 5.3 ± 2.6 in metformin non-users, respectively.

At the end of the follow-up period, 95 (12.2%) of 778 metformin users and 256 (16.6%) of 1546 non-users developed ESKD requiring renal replacement therapy (incidence rates of 2285 vs. 3136.2 events per 1,000,000 patient-years) (Table 2). Compared to non-users, the metformin users had a significantly lower crude hazard ratio (0.72, 95% CI 0.56–0.93, *p* = 0.01) for ESKD. The association remained unchanged after fully adjusting for baseline covariates (HR: 0.75, 95% CI 0.58–0.97; *p* = 0.03). After considering the competing risk of death before renal outcomes, metformin users also had a significantly lower hazard of renal outcomes than metformin non-users (HR: 0.71, 95% CI 0.56–0.89, *p* = 0.03). Similarly, a decreased hazard of MACEs was noted in the metformin users, with an adjusted HR of 0.78 (95% CI 0.65–0.95, *p* = 0.01). The separate events for MACEs are shown in Table S3. However, there was no significant difference in the hazards of all-cause mortality between the two groups. Regarding the intensity of metformin use, this dose–response relation was not noted for ESKD, MACE, and mortality (Table S4). When assessing the effect of the severity of DM on the main results, metformin users treated with and without insulin both had significantly lower ESKD hazards (Table S5). The hazard of MACEs decreased significantly in those with insulin use, while the hazard of all-cause mortality decreased significantly in those without insulin use (Table S5).

Similar analyses were performed in patients with both DM and ADPKD diagnoses (Table 1 and Table 2). Of 9742 patients in the study cohort, 1313 had diagnoses of both DM and ADPKD. Among them, 741 patients were metformin users and 572 were non-users. After matching participants in a 1:1 ratio, the groups of metformin users and non-users had balanced covariate distributions. During this period, 40 (15.3%) of the 261 metformin users and 76 (29.1%) of 261 non-users developed ESKD (incidence rates of 2597.5 vs. 5539.9 events per 1,000,000 patient-years). The results of the analysis of the associations between metformin use and clinical outcomes were consistent with the main findings. The metformin users still demonstrated a lower hazard of ESKD (HR:0.35, 95% CI 0.20–0.59, *p* < 0.0001) and MACEs (HR: 0.72, 95% CI 0.53–0.09, *p* < 0.0001).

The sensitivity analysis was performed by stratifying patients into stage 5 and other stages (Table 3). The decreased hazard ratios of metformin use for ESKD (HR: 0.78, 95% CI 0.60–1.02, *p* = 0.07 and MACE (HR: 0.81, 95% CI 0.66–1.00, *p* = 0.05) were observed in other CKD stages after excluding CKD stage 5. The associations were more obvious among patients having diagnoses of both ADPKD and DM, with HRs of 0.41 (95% CI 0.26–0.64, *p* < 0.0001) for ESKD and 0.66 (95% CI 0.47–0.94, *p* = 0.02) for MACEs. Conversely, the hazards tended to increase in stage 5 CKD, although this association did not reach statistical significance.

**Figure 2:**
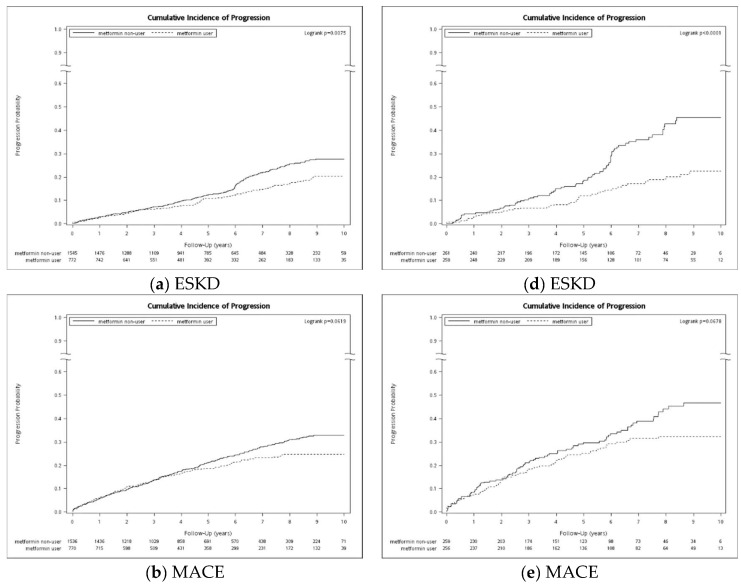

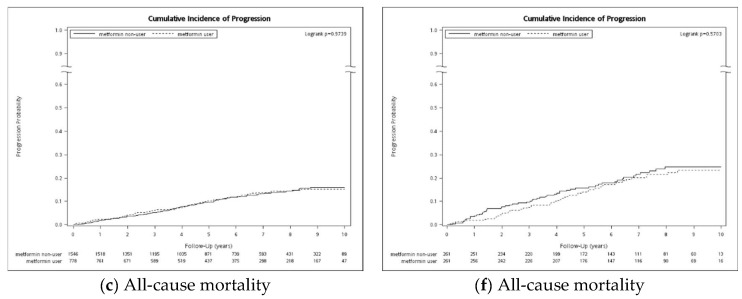
Kaplan–Meier analysis for (**a**) ESKD, (**b**) MACE, and (**c**) all-cause mortality among matched ADPKD cohort according to metformin use. Kaplan–Meier analysis for (**d**) ESKD (**e**) MACE (**f**) all-cause mortality among matched cohort with diagnoses of both ADPKD and DM, according to metformin use. Abbreviations: ADPKD: autosomal dominant polycystic kidney disease. DM: diabetes mellitus. ESKD: end-stage kidney disease. MACE: major adverse cardiovascular event.

The cumulative incidences of ESKD and MACEs also showed a lower cumulative incidence in the metformin user group compared to non-users (Figure 2). The Log-Rank test demonstrated a significantly lower cumulative incidence of ESKD in patients diagnosed with ADPKD or both ADPKD and DM.

**Figure 3:**
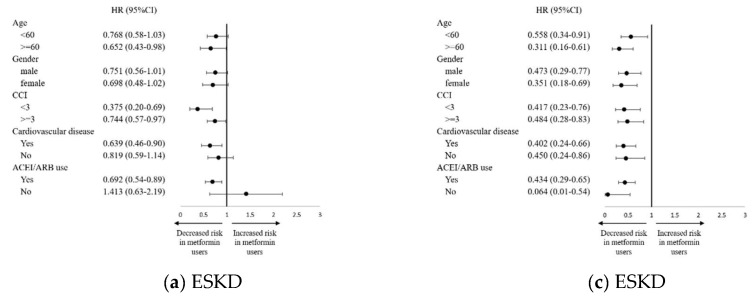

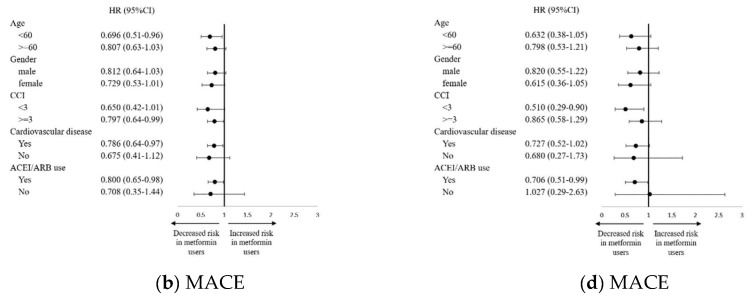
Risk for ESKD and MACE by metformin use in subgroup analysis. Hazard ratios of (**a**) ESKD and (**b**) MACE among matched patients with ADPKD. (**c**) ESKD (**d**) MACE among matched patients with diagnoses of both ADPKD and DM. Abbreviations: ESKD: end-stage kidney disease. MACE: major adverse cardiovascular event. ADPKD: autosomal dominant polycystic kidney disease. CCI: Charlson comorbidity index. ACEI: angiotensin-converting enzyme inhibitor. ARB: angiotensin receptor blocker.

Subgroup analyses indicated that metformin users appeared to have significantly decreased hazards of ESKD, among patients with ADPKD who were aged ≥60, taking ACEI/ARB, had CCI ≥ 3, or had cardiovascular disease (*p* for interaction = 0.03, 0.91, <0.001, 0.94, respectively) (Figure 3). However, the effects of metformin use on ESKD were consistent with the main findings across all clinically relevant subgroups among patients with both ADPKD and DM (all *p* values for interaction ≥ 0.05). As for MACEs, the hazard was significantly lower in metformin users, especially in patients with ADPKD aged < 60, who were taking ACEI/ARB, who had CCI ≥ 3, or who had cardiovascular disease (*p* values for interaction = 067, 0.68, 0.13, <0.0001, respectively). There was a decreased hazard for those patients diagnosed with ADPKD and DM with CCI < 3 and who were taking ACEI/ARB (*p* values for interaction = 0.02 and 0.12, respectively).

## 4. Discussion

This nationwide population-based cohort study demonstrated that metformin use was associated with significantly lower hazards of ESKD and MACEs after adjustment for potential confounding effects in ADPKD patients with a combined diagnosis of DM or not. These findings were consistent after sensitivity analyses by excluding late-stage CKD (CKD stage 5 or ESA use). Nevertheless, the hazards of all-cause mortality did not differ between the metformin user and non-user groups. Importantly, this is the first nationwide cohort study to support the protective role of metformin treatment in reducing hazards of kidney outcomes and MACE events among ADPKD patients.

ADPKD is characterized by the development of multiple cysts expanding throughout the kidney parenchyma. Cystogenesis and marked kidney volume increase over many decades and result in gradual kidney function decline and eventually ESKD, which accounts for 5–10% of ESKD worldwide [24,25]. However, the clinical phenotype of ADPKD is highly variable due to the presence of heterogeneous genetic mutations or environmental modifiers [26,27,28]. Specifically, mutations in PKD2 versus PKD1 have been confirmed to present favorable kidney prognoses, with an average age at ESKD of 79 and 58 years, respectively [29,30]. Additional genetic factors that determine the time of disease onset or kidney function progression include allelic mutations (truncating, non-truncating, or hypomorphic), mosaicism, and other cystic-related gene mutations [7,31,32,33]. In Taiwan, ADPKD contributes to only 2.25% of the dialysis population [34]. Recently, the database of the Taiwan PKD Consortium has provided the mutation spectrum indicating that a high frequency of a single PKD2 p.Arg803* mutation (17.8% of the cohort) was found with relatively slower kidney function decline [35].

PC1 and PC2, mainly expressed on the primary cilia, are products of PKD1 and PKD2 genes that modulate several signaling pathways and inhibit cystogenesis involving intracellular calcium control [36,37,38]. A series of studies have also demonstrated that reduced intracellular calcium in cystic epithelium contributes to intracellular cAMP accumulation, which enhances protein kinase A (PKA)-dependent gene transcription, subsequently promoting epithelial cell proliferation by activation of the MEK/ERK pathway [39,40]. Accordingly, treatment with cAMP analogs increased chloride transport through the CFTR and terminated in fluid secretion and cyst expansion [41,42]. Moreover, PC1 interacts with tuberous sclerosis complex proteins 2 that increase mTOR signaling and regulate cell growth [43,44]. Therefore, some important advances in ADPKD treatment focused on cAMP inhibition have been reported to slow the progression of PKD [45,46].

Metformin has been known as a stimulator of AMPK, which serves as a cellular energy-sensing molecule that inhibits the CFTR channels and mTOR pathways during energy depletion. Growing evidence has indicated dysregulated cellular metabolism (increased aerobic glycolysis) in PKD pathogenesis. Lian et al. reported that the combination treatment of metformin and 2-deoyglucose (a glycolytic inhibitor) in a miniature pig model suppressed PKA, mTOR, and ERK and activated AMPK, and accordingly retarded kidney function progression [47,48]. In another pkd1 mouse model, the administration of metformin lowered the cystic index and showed modest effects on cyst growth without reverting cystogenesis [17]. Furthermore, Chang et al. demonstrated that metformin treatment prevented cyst formation by activating AMPK and modulating proliferation and autophagy in the pkd2 zebrafish model [18].

The clinical effects of metformin in ADPKD have been investigated in a small number of clinical trials, and a phase 3 clinical trial is ongoing [49]. The METROP study, the first prospective and single-arm study, showed a good safety profile without any change in eGFR during the 2-year follow-up period in 34 ADPKD patients with stages 1–5 CKD [50]. In a small-scale randomized controlled study, 500–1000 mg of metformin twice daily was well tolerated over 12 months in 51 ADPKD participants with an eGFR of 50–80 mL/min/1.73 m2 without significant changes in height-adjusted total kidney volume (htTKV) or eGFR [51]. Moreover, the phase 2 Trial of Administration of Metformin in PKD (TAME PKD) study showed that eGFR decline and htTKV growth were numerically less, but not significantly so, with metformin because the study was not significantly powered to detect such differences [20]. A retrospective database analysis of seven diabetic ADPKD patients with stage 3 CKD treated with metformin and seven matched non-diabetic ADPKD patients demonstrated that metformin had a beneficial effect on delaying kidney function progression during the 3-year follow-up period [52]. More recently, a systemic review reported that compared to placebo, metformin may have the effect of a slight decline in kidney function (three studies, 505 participants: MD 1.92 mL/min, 95% CI 0.33 to 3.51; I^2^ = 0%) but an uncertain effect on ESKD (one study, 753 participants: RR 1.20, 95% CI 0.17 to 8.49) [53]. Our study suggests that metformin reduces the hazard of ESKD among ADPKD patients, consistent with the concept that metformin might exert renoprotective properties on ADPKD progression. Nevertheless, in late-stage CKD (CKD stage 5), we observed the trend of increasing risk of ESKD, although it did not reach significance. The lack of beneficial impact on ESKD risk might imply that the experimental reduction in cytogenesis or kidney function decline may not reverse disease progress since the late-stage CKD was advanced.

Cardiovascular complications, such as HTN, intracranial aneurysm, valvular heart disease, and left ventricular hypertrophy, are the leading cause of morbidity and mortality in ADPKD patients. The driving factors of CVD include angiotensin–aldosterone system (RAAS) and sympathetic nerve system overactivity due to enlarging cysts, or ciliary-related abnormalities in vascular endothelial cells, smooth muscle cells, and cardiomyocytes [5,54]. Although ADPKD-specific CVD could precede clinical kidney disease, there is limited literature reporting the optimal control of CV events in ADPKD. To date, the NHIRD data in Taiwan showed that both RAAS blockade and statin use reduced cerebrovascular events in ADPKD without dialysis [55]. Considering the role of metformin on CV outcome in CKD, prior studies presented conflicting results on CVD protection in stages 3–4 CKD [56,57]. Our study is the first to indicate the protective effect of metformin use on MACE outcomes in ADPKD. Similar to the ESKD outcome, the survival benefits disappeared in patients with advanced CKD stage in our sensitivity test, corroborating Hung’s findings of a dose-dependent higher risk of mortality in advanced CKD [58]. Furthermore, the clear effects of metformin on ADPKD-specific cardiovascular pathways and endothelial dysfunction require more investigations.

Our study has some limitations. First, all the analyses retrieved from the NHIRD have inherent limitations due to the lack of laboratory data. Some potentially important confounders, such as blood pressure, body weight index, eGFR, lipid and sugar profiles, and inflammatory and nutritional status might affect the propensity score estimation and matching, as well as the relationship between metformin use and outcomes. Second, metformin treatment was based on prescription records; therefore, patient compliance was unknown. Third, although the disease definition depended on ICD codes validated in the NHIRD, coding errors, misclassification of CKD stages, or misdiagnosis of events may exist in database research. Moreover, the NHIRD released by the NHI Administration contains only the first five diagnosis codes. Therefore, the exact reasons why those ADPKD patients without DM diagnoses were prescribed metformin might include incomplete coding, prediabetes, PCOS, etc. All these issues might undermine the reliability of the findings, which should be interpreted with caution. Finally, the findings were generated from an Asian population; thus, they may not be generalizable to other races.

## 5. Conclusions

In conclusion, the population-based study findings suggest that metformin use is associated with a lower risk of ESKD and MACEs in ADPKD patients except those with advanced CKD (stage 5). Given that ADPKD is multifaceted, it is necessary to develop therapies targeting the retardation of cyst expansion and the regulation of vascular defects that can control cardiovascular complications. This study provides support for the idea that metformin might be a potential therapeutic option for mitigating kidney disease progression and reducing CVD risks in ADPKD. In the future, RCTs with sufficient statistical power are warranted to ascertain the causal association and assess the appropriate use in this population.

## Figures and Tables

**Table 1:** Baseline democratic and clinical characteristics by metformin usage status in propensity score-matched study cohorts.

	ADPKD			ADPKD + DM		
Variables	Metformin User	Metformin Non-User	*p* Value	Metformin User	Metformin Non-User	*p* Value
Number of subjects	778	1546		261	261	
Age (mean ± SD)	59.5 (13.9)	59.3 (14.4)		62.6 (12.7)	62.6 (12.4)	
Age groups			1.00			1.00
<40	71 (9.1%)	142 (9.2%)		7 (2.7%)	7 (2.7%)	
40–49	117 (15.0%)	234 (15.1%)		32 (12.3%)	32 (12.3%)	
50–59	209 (26.9%)	414 (26.8%)		78 (29.9%)	78 (29.9%)	
60–69	199 (25.6%)	392 (25.4%)		68 (26.1%)	68 (26.1%)	
>70	182 (23.4%)	364 (23.5%)		76 (29.1%)	76 (29.1%)	
Index year			1.00			1.00
2009–2013	420 (54%)	840 (54.3%)		176 (67.4%)	176 (67.4%)	
2014–2018	358 (46%)	706 (45.7%)		85 (32.6%)	85 (32.6%)	
Gender (male)	478 (61.4%)	956 (61.8%)	0.85	177 (67.8%)	177 (67.8%)	1.00
Hypertension	696 (89.5%)	1376 (89%)	0.74	230 (88.1%)	244 (93.5%)	0.03
Dyslipidemia	587 (75.5%)	1151(74.5%)	0.60	194 (74.3%)	183 (70.1%)	0.28
Gout	279 (35.9%)	595 (38.5%)	0.22	111 (42.5%)	124 (47.5%)	0.25
Cardiovascular disease	371 (47.7%)	695 (44.9%)	0.21	165 (63.2%)	155 (59.4%)	0.37
Peripheral artery disease	76 (9.8%)	137 (8.9%)	0.48	40 (15.3%)	36 (13.8%)	0.62
Cerebrovascular disease	279 (35.9%)	595 (38.5%)	0.22	111 (42.5%)	124 (47.5%)	0.25
CCI score			0.88			1.00
CCI < 3	232 (29.8%)	685 (44.3%)		34 (13.0%)	28 (10.7%)	
CCI 3–4	257 (33.0%)	586 (37.9%)		105 (40.2%)	82 (31.4%)	
CCI ≧ 5	289 (37.2%)	275 (17.8%)		122 (46.7%)	151 (57.9%)	
Mean	3.9 (2.3)	3.0 (1.8)		4.7 (2.1)	4.9 (2.0)	
Antihypertensive drugs						
ACEI/ARB	676 (86.9%)	1274 (82.4%)	0.006	227 (87.0%)	230 (88.1%)	0.691
CCB	761 (97.8%)	1506 (97.4%)	0.554	253 (96.9%)	252 (96.6%)	0.805
Beta blockers	772 (99.2%)	1517 (98.1%)	0.039	257 (98.5%)	257 (98.5%)	1.000
Others	632 (81.2%)	1199 (77.6%)	0.041	217 (83.1%)	212 (81.2%)	0.567
Numbers of anti-HTN drug			0.02			0.15
1	340 (43.7%)	683 (44.2%)		110 (42%)	110 (42%)	
2	244 (31.4%)	512 (33.1%)		78 (29.7%)	72 (27.4%)	
3	150 (19.3%)	253 (16.4%)		51 (19.4%)	52 (20.1%)	
4	44 (5.6%)	98 (6.3%)		22 (8.9%)	27 (10.3%)	
CKD stage 5 or ESA use	67 (8.6%)	260 (16.8%)	<0.001	29 (11.1%)	89 (34.1%)	<0.001
Follow-up years	5.8 (2.9)	5.8 (2.9)	0.38	6.5 (2.7)	6.2 (2.8)	0.54

Abbreviations: ADPKD: autosomal dominant polycystic kidney disease. DM: diabetes mellitus. CCI: Charlson comorbidity index. ACEI: angiotensin-converting enzyme inhibitor. ARB: angiotensin receptor blocker. CCB: calcium channel blocker. CKD: chronic kidney disease. ESA: erythropoiesis stimulating agent. Data are presented as mean (standard error) or count (percentage%).

**Table 2:** Association between metformin usage status and clinical outcomes in ADPKD cohort.

	ADPKD			ADPKD + DM		
	Metformin Non-User	Metformin User	*p*	Metformin Non-User	Metformin User	*p*
Number of subjects	1546 (66.5%)	778 (33.5%)		261 (50%)	261 (50%)	
**ESKD**						
No of events	256 (16.6%)	95 (12.2%)		76 (29.1%)	40 (15.3%)	
Incidence rate	3136.2	2285.0		5539.9	2597.5	
Unadjusted HR	1.00 (reference)	0.72 (0.56–0.93)	0.01	1.00 (reference)	0.40 (0.26–0.63)	<0.0001
Fully adjusted HR	1.00 (reference)	0.75 (0.58–0.97)	0.03	1.00 (reference)	0.35 (0.20–0.59)	<0.0001
Competing risk	1.00 (reference)	0.71 (0.56–0.89)	0.003	1.00 (reference)	0.47 (0.32–0.68)	<0.0001
**MACE**						
No of events	349 (22.6%)	147 (18.9%)		89 (34.1%)	71 (27.2%)	
Incidence rate	4568.3	3773.3		6991.3	5109.6	
Unadjusted HR	1.00 (reference)	0.88 (0.72–1.08)	0.22	1.00 (reference)	0.62 (0.43–0.89)	0.01
Fully adjusted HR	1.00 (reference)	0.78 (0.65–0.95)	0.01	1.00 (reference)	0.72 (0.53–0.99)	0.04
**Death**						
No of events	171 (11.1%)	86 (11.0%)		53 (20.3%)	49 (18.8%)	
Incidence rate	1905.5	1912.5		3253.4	2907.3	
Unadjusted HR	1.00 (reference)	1.01 (0.75–1.37)	0.94	1.00 (reference)	0.58 (0.36–0.95)	0.03
Fully adjusted HR	1.00 (reference)	0.99 (0.76–1.28)	0.94	1.00 (reference)	0.90 (0.60–1.34)	0.60

Fully adjusted model: adjusted for age, sex, Charlson comorbidity index score, gout, hypertension, and all covariates. Incidence rate: per 1,000,000 patient-years. Abbreviations: ADPKD: autosomal dominant polycystic kidney disease. DM: diabetes mellitus. ESKD: end-stage kidney disease. MACE: major adverse cardiovascular event. HR: hazard ratio.

**Table 3:** Hazard ratios of metformin usage status and clinical outcomes by different CKD stages.

	ADPKD			ADPKD + DM		
	Metformin Non-User	Metformin User	*p*	Metformin Non-User	Metformin User	*p*
**CKD stage 5 or ESA use**						
ESKD	1.00 (reference)	1.36 (0.77–2.39)	0.29	1.00 (reference)	1.61 (0.73–3.56)	0.24
MACE	1.00 (reference)	1.19 (0.60–2.34)	0.62	1.00 (reference)	1.56 (0.62–3.92)	0.35
Death	1.00 (reference)	1.95 (0.93–4.10)	0.08	1.00 (reference)	1.92 (0.60–6.20)	0.27
**Other CKD stages**						
ESKD	1.00 (reference)	0.78 (0.60–1.02)	0.07	1.00 (reference)	0.41 (0.26–0.64)	<0.0001
MACE	1.00 (reference)	0.81 (0.66–1.00)	0.05	1.00 (reference)	0.66 (0.47–0.94)	0.02
Death	1.00 (reference)	1.02 (0.76–1.35)	0.92	1.00 (reference)	0.93 (0.59–1.45)	0.74

Fully adjusted model: adjusted for age, sex, Charlson comorbidity index score, gout, hypertension, all covariates. Abbreviations: ADPKD: autosomal dominant polycystic kidney disease. DM: diabetes mellitus. ESKD: end-stage kidney disease. MACE: major adverse cardiovascular event. CKD: chronic kidney disease.

## Data Availability

All data are included in the manuscript and/or supporting information for reference. The raw data could only be accessed and analyzed under the regulations of the Health and Welfare Data Science Centre, Ministry of Health and Welfare. Further inquiries can be directed to the corresponding author.

## Supplementary Materials

The following supporting information can be downloaded at https://www.mdpi.com/article/10.3390/biomedicines13030635/s1. Table S1: ICD9 code and ICD10 code used in this study; Table S2: Baseline characteristics before matching; Table S3: Association between metformin usage status and separate events of MACE; Table S4: Dose–response analysis for the effect of metformin use in ADPKD; Table S5: Association between metformin use and clinical outcomes stratified by insulin usage in ADPKD cohort.
