# Supplementary material for: Metformin Use and Clinical Outcomes in Autosomal Dominant Polycystic Kidney Disease: A Nationwide Cohort Study
Source: Biomedicines. 2025 Mar 5;13(3):635. doi: 10.3390/biomedicines13030635 (PMC11940305; doi:10.3390/biomedicines13030635)
Supplement: Supplementary file 1 [file biomedicines-13-00635-s001.zip › biomedicines-3444361-supplementary.pdf]

Supplement Table S1 ICD9-code and ICD10-code used in this study.

Disease	ICD9-code	ICD10-code
**Comorbidities**		
Hypertension	401-405	I10-I16
Dyslipidemia	272	E78
Gout	274	M10, M1A
Cardiovascular disease	410-414,428, 402.01, 402.11, 402.91, 404.01, 404.03, 404.11, 404.13, 404.91, 404.93	M30-M36
Cerebrovascular disease	430-438	G45-G46, I60-I69
Peripheral arterial disease (PAD)	440.2, 440.3, 440.8, 440.9, 443, 444.22, 444.8, 444.9	I70.2-I70.9, I73.9 I74.3, I74.9, I75.029
Diabetes Mellitus	249-250	E08-E13
Malignancy	140-208	C00-C99, D00-D09
**Major adverse cardiovascular events (MACEs)**		
Acute coronary syndrome	410,411	I20-I22
Heart failure (HF)	428	I50
Acute ischemic stroke	433-437	I63, I65, I66, I67, G45
Renal replacement therapy (RRT)		
End-stage renal disease (ESRD)	585	N186
Kidney transplant	55.69	Z94.0

Supplement Table S2 Baseline characteristics before matching

	ADPKD(before matching)			ADPKD+DM(before matching)		
**Variables**	**Metformin user**	**Metformin nonuser**	**P value**	**Metformin user**	**Metformin nonuser**	**P value**
Number of Subjects	778	8964		741	572	
Age (Mean±SD)	59.5 (13.9)	52.8 (15.9)	<.0001	60.2 (13.7)	62.3 (13.7)	0.01
Age groups			<.0001			0.11
<40	71 (9.1%)	2102 (23.4%)		57 (7.7%)	37 (6.5%)	
40-49	117 (15.0%)	1874 (20.9%)		107 (14.4%)	65 (11.4%)	
50-59	209 (26.9%)	2147 (24.0%)		201 (27.1%)	139 (24.3%)	
60-69	199 (25.6%)	1420 (15.8%)		194 (26.2%)	162 (28.3%)	
>70	182 (23.4%)	1421 (15.9%)		182 (24.6%)	169 (29.6%)	
Index year						
2009-2013	420 (54%)	5236 (58.4%)		400 (54.0%)	408 (71.3%)	
2014-2018	358 (46%)	3728 (41.6%)		341 (46.0%)	164 (28.7%)	
Gender (male)	478 (61.4%)	4797(43.5%)	<.0001	459 (61.9%)	346 (60.5%)	0.59
Hypertension	696 (89.5%)	6789 (75.7%)	<.0001	665 (89.7%)	507 (88.6%)	0.52
Dyslipidemia	587 (75.5%)	3794 (42.3%)	<.0001	568 (76.7%)	323 (56.5%)	<.0001
Gout	279 (35.9%)	2487 (27.7%)	<.0001	270 (36.4%)	251 (43.9%)	0.01
Cardiovascular disease	371 (47.7%)	2990 (33.4%)	<.0001	360 (48.6%)	343 (60.0%)	<.0001
Peripheral artery disease	76 (9.8%)	722 (8.1%)	0.09	75 (10.1%)	108 (18.9%)	<.0001
Cerebrovascular disease	279 (35.9%)	2487 (27.7%)	<.0001	270 (36.4%)	251 (43.9%)	0.01
CCI score			<.0001			0.48
CCI<3	232 (29.8%)	3442 (38.4%)		184 (24.8%)	128 (22.4%)	
CCI 3-4	257 (33.0%)	2698 (30.1%)		262 (35.4%)	198 (34.7%)	
CCI≧5	289 (37.2%)	2824 (31.5%)		295 (39.8%)	245 (42.9%)	
Mean	3.9 (2.3)	2.3 (2.0)		4.0 (2.3)	4.9 (2.3)	
Antihypertensive drugs						
ACEI/ARB	676 (86.9%)	7700 (85.9%)	0.01	646 (87.2%)	500 (87.5%)	0.94
CCB	761 (97.8%)	8641 (96.4%)	0.01	720 (97.3%)	557 (97.4%)	0.89
Beta blockers	772 (99.2%)	8820 (98.4%)	0.01	732 (98.9%)	564 (98.7%)	0.93
Others	632 (81.2%)	7180 (90.1%)	<.0001	610 (82.4%)	474 (82.9%)	0.81
CKD Stage 5 or ESA use	67 (8.6%)	1300 (14.5%)	< 0.001	67 (9.0%)	210 (36.7%)	<0.001
Follow up year	5.8 (2.9)	6.3 (1.7)	0.07	6.4 (2.3)	6.3(2.7)	0.57

Supplement Table S3 Association between metformin usage status and separate events of MACE.

	ADPKD			ADPKD+DM		
	**Metformin nonuser**	**Metformin user**	***P***	**Metformin nonuser**	**Metformin user**	***P***
Number of subjects	1546(66.5%)	778(33.5%)		261(50%)	261(50%)	
**Acute ischemic stroke**						
No of events	149(9.6%)	64(8.2%)		38(14.6%)	32(12.3%)	
Incidence rate	1777.5	1526.5		2568.7	2074.3	
Fully-adjusted HR	1.00 (reference)	0.85 (0.64-1.14)	0.29	1.00 (reference)	0.81 (0.50-1.29)	0.37
**Acute coronary syndrome**						
No of events	137(8.9%)	55(7.1%)		36(13.8%)	29(11.11%)	
Incidence rate	1610.5	1281.6		2408.6	1856.3	
Fully-adjusted HR	1.00 (reference)	0.72 (0.52-0.98)	0.04	1.00 (reference)	0.75 (0.46-1.23)	0.25
**Acute heart failure**						
No of events	131(8.5%)	58(7.5%)		39(15.0%)	30(11.5%)	
Incidence rate	1542.4	1345.2		2648.0	1890.0	
Fully-adjusted HR	1.00 (reference)	0.84 (0.61-1.14)	0.25	1.00 (reference)	0.68 (0.42-1.09)	0.11

Fully-adjusted model: adjusted for age, sex, CCI score, gout, hypertension, all covariants

Incidence rate: per 1000000 patient-years

Abbreviations: HR: hazard ratio.

Supplement Table S4 Dose-response analysis for the effect of metformin use in ADPKD

	Metformin nonuser	DDD <7	DDD 7-12	DDD >12
**Total N**	1546	210	207	361
**ESKD**				
No of events	256(16.6%)	35(16.7%)	14(6.8%)	46(12.7%)
Incidence rate	8126.7	1106.9	1116.5	1934.2
Unadjusted HR	1.00 (reference)	0.76 (0.55-1.03)	0.40 (0.23-0.68)	1.00 (0.71-1.43)
Fully-adjusted HR	1.00 (reference)	0.74 (0.54-1.01)	0.44 (0.26-0.75)	0.96 (0.67-1.36)
**MACE**				
No of events	349(22.6%)	44(20.9%)	37(17.8%)	66(18.3%)
Incidence rate	7639.5	1007.4	1038.5	1849.8
Unadjusted HR	1.00 (reference)	0.79 (0.61-1.03)	0.78 (0.56-1.10)	0.96 (0.70-1.32)
Fully-adjusted HR	1.00 (reference)	0.82 (0.63-1.07)	0.73 (0.52-1.03)	0.78 (0.57-1.07)
**All-cause mortality**				
No of events	171(11.1%)	44(20.9%)	13(6.3%)	29(8.0%)
Incidence rate	8974.3	1224.9	1165.8	2105.9
Unadjusted HR	1.00 (reference)	0.72 (0.49-1.07)	0.58 (0.33-1.02)	1.90 (1.36-2.64)
Fully-adjusted HR	1.00 (reference)	0.79 (0.53-1.17)	0.59 (0.33-1.03)	1.60 (1.14-2.24)

Fully-adjusted model: adjusted for age, sex, CCI score, gout, hypertension, all covariants

Incidence rate: per 1000000 patient-years

Abbreviations: HR: hazard ratio.

Supplement Table S5 Association between metformin user and clinical outcomes stratified by insulin usage in ADPKD cohort

	**With insulin**			**Without insulin**		
	Metformin nonuser	Metformin user	*P*	Metformin nonuser	Metformin user	*P*
**ESKD**						
No of events	37(33.9%)	39(18.2%)		219(15.2%)	56(9.9%)	
Incidence rate	7998.6	3683.3		2844.1	1807.2	
Unadjusted HR	1.00 (reference)	0.44 (0.28-0.69)	<.0001	1.00 (reference)	0.63 (0.47-0.85)	0.002
Fully-adjusted HR	1.00 (reference)	0.46 (0.29-0.73)	0.002	1.00 (reference)	0.64 (0.48-0.86)	0.003
**MACE**						
No of events	45(41.3%)	64(29.9%)		304(21.2%)	83(14.7%)	
Incidence rate	11073.1	6725.1		4202.9	2819.2	
Unadjusted HR	1.00 (reference)	0.63 (0.44-0.93)	0.02	1.00 (reference)	0.68 (0.53-0.86)	0.002
Fully-adjusted HR	1.00 (reference)	0.76 (0.51-1.14)	0.19	1.00 (reference)	0.64 (0.50-0.82)	<.0001
**All-cause mortality**						
No of events	45(41.3%)	47(21.9%)		126(8.8%)	39(6.9%)	
Incidence rate	7861.2	3930.2		1499.7	1181.5	
Unadjusted HR	1.00 (reference)	0.50 (0.33-0.76)	0.001	1.00 (reference)	0.79 (0.55-1.13)	0.19
Fully-adjusted HR	1.00 (reference)	0.43 (0.27-0.67)	<.0001	1.00 (reference)	0.83 (0.58-1.18)	0.29

Fully-adjusted model: adjusted for age, sex, CCI score, gout, hypertension, all covariants

Incidence rate: per 1000000 patient-years

Abbreviations: HR: hazard ratio.
